# Self-diagnosis & pain management in dental students in Riyadh, KSA

**DOI:** 10.11604/pamj.2019.34.198.18347

**Published:** 2019-12-16

**Authors:** Shahad Mohammed Halawani, Lingam Amara Swapna, Sahar Amer Al-Harbi, Burhan Nezar Hamdi, Farah Masaad, Pradeep Koppolu

**Affiliations:** 1 Department of Oral Medicine and Diagnostic Sciences, Al Farabi Dental College, Riyadh, KSA; 2 Department of Surgical & Diagnostic Sciences, College of Dentistry, Dar Al Uloom University, Riyadh, KSA; 3 Department of Preventive Dental Science, College of Dentistry, Dar Al Uloom University, Riyadh, KSA

**Keywords:** Self-diagnosis, pain management, internet

## Abstract

**Introduction:**

Self-diagnosis and pain management is a worldwide practice. The current study aims to determine the percentage of dental students and interns who self-diagnose and manage their dental pain and further establish the proportion of students who depend on various resources for diagnosing and treating their condition.

**Methods:**

A cross-sectional, self-administered questionnaire-based study was conducted among the dental students in and around Riyadh. The questionnaire consisted of three parts including: part 1-demographic data; part 2-pain and self-diagnosis; part 3-visiting the dentist and managing the pain. The data were analyzed using the Statistical Package for Social Sciences (SPSS version 22.0).

**Results:**

Fifty four percent of the participants were involved in self-diagnosis and managed the pain by themselves. Seventy three percent of the respondents experienced teeth/gum discomfort or any symptoms of an oral health problem, of which 57% searched the symptoms they faced on the internet to arrive at a diagnosis. Besides, 35% of the interns considered internet to be a helpful tool for diagnosing their pain. 16% admitted that they have never visited a dentist.

**Conclusion:**

We found that a significant proportion of the participants self-diagnosed by using their background or resorting to the internet, at times consulting a dentist to confirm their diagnosis. The students from the health sciences background should refrain from this practice. Efforts should be made to make the population mindful of the potential risks linked to self-medication and diagnosis. Further research should be done with a larger sample size by including the students and interns from different institutions.

## Introduction

The International Association for the Study of Pain (IASP) describes pain as an unpleasant sensory and emotional experience associated with actual or potential tissue damage [1]. Pain is not just a corporeal sensation but is prejudiced by attitude, opinion, character and communal factors, and the problem can affect the emotional and psychological wellbeing of an individual. Although two people may have the same pain, their capability of living with it can be vastly different. Classically, pain can be categorized as acute, chronic and cancer-related pain [1,2]. Discomfort experienced in the form of pain can be helpful in identifying the underlying health problem. Pain is one of the most common reasons for individuals to seek dental advice. Unrelieved pain can have an adverse impact on the student's quality of life, yet some dental students do not seek any dental advice and choose to self-diagnose and manage their pain by themselves. The dental undergraduates are strongly tempted to contemplate on their symptoms and arrive at their own conclusions. When the students indulge in self-diagnosis, they are basically assuming that they are aware of the subtleties that diagnosis entails. The treasure of consistent online health information often makes these students feel comfortable while searching their symptoms. However, this practice could cause serious health hazards as people who assume that they can surmise what is going on with them may miss the nuances of diagnosis [3-6]. Dental pain can arise as a result of any of the following underlying issues: dental decay, periodontal disease, abscess, sinusitis, referred TMJ pain and wisdom teeth. One of the dangers associated with self-diagnosis is managing the pain with the wrong method owing to incorrect diagnosis. Self-medication has conventionally been defined as "the consuming of drugs, herbs or home remedies on one's own initiative, or on the advice of another person, without consulting a doctor" [2,3]. Families, friends, neighbors, the pharmacist, previously prescribed drugs, and suggestions from advertisements in newspapers or popular magazines are the common sources of self-medications. This habit could be perceived as the "desire and talent of people/patients to play an intelligent, independent and informed role, not merely in terms of decision-making but also in the management of those preventive, diagnostic and therapeutic activities which concern them". The major complications related to self-medication are depletion of resources, increased resistance to pathogens, and serious health hazards such as adverse reaction and persistent suffering [3-5].

## Methods

A cross-sectional, self-constructed questionnaire was distributed randomly among the dental students (third year onwards) and interns (n=505) in and around Riyadh, KSA (kingdom of Saudi Arabia) between March 2017 and June 2017. The questionnaire comprised 11 questions and was organized in three parts. Part 1: questions related to demographic information. Part 2: questions related to pain and self-diagnosis. Part 3: questions related to visiting the dentist and managing the pain. The forms were personally distributed to the individual students and the web link was also provided through mail and social media. Instructions for filling the questionnaire were given, and the students were informed that their participation was purely voluntary. The content authenticity was pretested on a random sample of population to ascertain practicability, strength and interpretation of the answers. Those who refused to fill the questionnaire were excluded from our study. Ethical clearance was obtained from the Ethical Committee of Al Farabi Dental College. The students who were willing to participate were included in the study, and their consent signature was obtained in the same form. The data were entered in the Statistical Package for Social Sciences (SPSS22.0), analyzed and interpreted using descriptive statistics (calculation of frequency, percentage, median, mean, mode, etc.).

## Results

A total of 505 students and interns in and around Riyadh, KSA participated in our study and over 70% of them reported tooth/gum discomfort or any symptoms of an oral health problem. Among the respondents, 57% were females and the other 43% were males (Table 1). The highest response rate of 25% was obtained for the 6th year students, followed by the interns (24%), 4^th^ year (19%), 5^th^ year (19%) and 3^rd^ year dental students (13%) (Table 1). It was discerned that 73% of the respondents experienced discomfort in their teeth or gums or faced any symptoms of an oral health problem, while 26% had never encountered such an issue. The findings indicated that 79% had diagnosed their dental pain on their own, and only 21% did not indulge in self-diagnosis. Furthermore, 57% searched the symptoms they faced on the internet to arrive at a diagnosis, while the remaining 43% refrained from such an action (Table 2). Besides, 62% of the respondents confessed that they confirmed the self-diagnosis with a dentist and found it to be correct, 9% came to know that they had incorrectly diagnosed the condition, 16% had not visited the dentist for their dental problem, and 12% had not diagnosed themselves (Table 2). The results pointed that 42% of those who did not visit the dentist managed the pain by themselves, 27% did not manage the pain, and the other 30% visited the dentist (Table 3). Analysis of the data also suggested that 43% postponed the visit to the dentist by changing their eating habits, consuming soft diet, etc. while 55% made no such attempts (Table 3). Moreover, 53% felt confident about managing their symptoms by any method without consulting a dentist, but 46% did not think that they can manage the pain by themselves (Table 4). An important detail that emerged from the study was that 35% of the participants considered the internet to be a helpful aid in getting a dental diagnosis. However, 15% did not agree about the usefulness of the internet, and 49% thought that it might sometimes be a helpful aid (Table 4). Among the respondents, 86% opined that the best way to manage dental pain was to consult a dentist, 10% supported the use of some medication, 2% were in favor of changing to soft diet, and 2% were ignorant of it (Table 5).

**Table 1:** Gender distribution and their academic year

Sex	Frequency	Valid percent
Female	287	56.8
Male	218	43.2
Total	505	100.0
**Academic year**	**Frequency**	**Valid percent**
3^rd^ year dental student	63	12.5
4^th^ year dental student (level 7 & 8)	98	19.4
5^th^ year dental student (level 11 & 12)	96	19.0
6^th^ year dental student (level 11 & 12)	127	25.2
Dental intern	120	23.8
Total	504	100.0
Unanswered	1	
Total	505	

**Table 2:** How students responded to the pain

Have you ever felt any discomfort with your teeth or gums or experienced any symptom of an oral health problem?	Frequency	Valid percent
Yes	370	73.6
No	133	26.4
Total	503	100.0
Unanswered	2	
Total	505	
**Have you searched the symptoms you faced on the internet to get to the diagnosis?**	**Frequency**	**Valid percent**
Yes		57.2
No	216	42.8
Total	505	100.0
**If you self-diagnosed yourself was your diagnosis correct after confirmation from a dentist?**	**Frequency**	**Valid percent**
Yes	314	62.5
No	44	8.8
I have not been to the dentist for my problem		
I have not diagnosed my self	83	16.5
Total	505	100.0

**Table 3:** Students visiting the dentist to diagnose tooth pain

Have you postponed your visit to dentist for pain by changing your eating habits or taking soft diet etc.?	Frequency	Valid percent
Yes	218	43.9
No	279	56.1
Total	497	100.0
Unanswered	8	
Total	**505**	
**If you have not visited the dentist for your self-diagnosed problem did you manage your pain by yourself?**	**Frequency**	**Valid percent**
Yes	210	42.1
No	138	27.7
I visited the dentist for my problem	151	30.3
Total	499	100.0
Unanswered	6	
Total	505	

**Table 4:** Students approach to tackle with tooth pain

Do you feel confident in managing your symptoms by any method without consulting a dentist?	Frequency	Valid percent
Yes	268	53.8
No	230	46.2
Total	498	100.0
Unanswered	7	
Total	505	
**In your opinion do you think the internet is a helpful aid in getting to a dental diagnosis?**	**Frequency**	**Valid percent**
Yes	177	35.3
No	76	15.2
Sometimes	248	49.5
Total	501	100.0
Unanswered	4	
Total	505	

**Table 5:** How students manage pain

In your opinion what is the best way to manage your Dental pain?	Frequency	Valid percent
Consulting a dentist	433	86.4
Pharmacologically	48	9.6
Changing to a soft diet	12	2.4
Ignorance	8	1.6
**Total**	501	100.0
Unanswered	4	
**Total**	505	

## Discussion

The aim of the study was to determine the percentage of dental students and interns who self-diagnose and manage dental pain by themselves, besides identifying the proportion of students who depend on internet to diagnose the condition. All the 505 dental students and interns who were surveyed belonged to hospitals and universities in Riyadh, Saudi Arabia. Previously, many researchers have documented the attitude of medical and dental students on the issue of self-medication. They noticed that the practice was prevalent among the medical and dental students as they tend to treat themselves for fever, headache, cough, common cold, acidity and diarrhea [7-10]. In a study carried out in India, it was observed that almost 97% of the dental students indulged in self-medication for minor health issues [7]. On the contrary, only 53% of the respondents in our study felt confident about managing their oral symptoms by any method without consulting a dentist. In a different study involving medical students in Ethiopia, it was observed that 64% of the students decided to proceed with their own choice of medicine for any minor health complaints and 43% practiced self-medication [8]. The rate of self-medication according to a study conducted in Iraq was 92.4% [9]. Similarly, it has been reported that 94% of the college students practiced self-medication in another study conducted in Oman [10]. The International Pharmaceutical Federation defines self-medication as the use of non-prescribed medications by public on their own initiative. In the developing countries, many people not only consume non-prescription drugs but also prescription drugs as self-medication products without supervision. This rise in self-care may be attributed to numerous factors such as socioeconomic issues, lifestyle, easy access to drugs, minor sickness, absence of escort, and lack of confidence in the prescribing doctor [11-13]. Not much research has been done particularly on dental students and their attitude towards managing oral disease or pain. The practice of self-medication was promoted by WHO for effective and quick relief of symptoms in rural and remote areas where healthcare services are understaffed and inaccessible [14].

In a study conducted in Malaysia on healthcare personnel, almost 78% of the participants were practicing self-medication, and majority of them were using it to treat mild illnesses such as cold, cough, fever, headache, etc. [15]. A study by Taniya Lewis in 2006 found that young people depended on online resources for their health issues. Most of the youngsters considered visiting their doctors as the first priority for medical concerns, but sought the web as a complementary rather than supplementary source of information [11]. Previous research among the university students in Islamabad implied that the prevalence of self-medication among the university students was 43% [16]. A few other studies on self-medication among the university students reported its prevalence to be 45% in Turkey, 88% in Croatia and 94% in Hong Kong [17-19]. Mike Benigeri, in his research, mentioned that there is a potential harm in internet-based information because of the existence of inaccurate and misleading content [6]. Survey findings advocate that web search may influence the anxiety levels and behaviors of those searching for information on undiagnosed conditions. Matthew S. Eastin concluded from his study that an individual's level of anxiety depends on their reliance on online health information and healthcare utilization decisions [20]. In another study, Baker *et al*. noticed that 40% of the respondents depended on the internet for seeking advice or information about health problems or healthcare. Nonetheless, 94% stated that the internet usage had no effect on the number of visits to the physician [12]. Though there are different sources of information such as elders´ advice, previous dental visit records, and the suggestions of experienced patients from which the students can be benefitted, 57% of them relied on the internet for any information. The results of another research among the university students in Bangladesh documented that 59% of the participants were using self-medication, with antipyretics and analgesics being the routinely used medications; however, only few of the students were aware of the side effects [21]. In an investigation involving the patients in the rural area of Maharashtra, India, a total of 52% of the respondents claimed that they were practicing self-medication [22], which indicates that people who are not highly educated are also habituated to its use. There is not much literature on how the dental students diagnose and manage their pain. Our study is the first of its kind to document the attitude of dental students in dealing with their oral health issues.

**Limitations:** the limitations of our research are the small sample size and the fact that it was conducted only in Riyadh, Saudi Arabia among the representatives of dental interns and 3^rd^-6^th^ year students. Besides, we need to consider the fluency in the English language, since the respondents were not native English speakers. A few participants responded to the questionnaire online through the survey link provided in e-mail or social media, and we cannot rely on the genuineness of their answers. Reliable data was not available owing to a lack of prior studies on this topic.

## Conclusion

This study surveyed male and female dental students and interns to ascertain whether they self-diagnose and manage the condition by themselves when encountering dental pain. We found that a significant proportion of the participants self-diagnosed by using their background or resorting to the internet, at times consulting a dentist to confirm their diagnosis. Others did not visit the dentist and managed the pain by themselves or changed their diet. However, the 87% agreed that the best way to manage the pain was to consult the dentist. Self-medication and diagnosis can indirectly lead to silent and serious complications. The students from the health sciences background should refrain from this practice and spread a word about its harmful effects to the others in the society, considering it as their social responsibility. Efforts should be made to make the population mindful of the potential risks linked to self-medication and diagnosis. Women should especially be educated about the implications of self-medication as using it during the reproductive period can have deleterious effects on the fetus. The results are based on the responses of the individuals who participated in the study and cannot be generalized. Further research should be done with a larger sample size by including the students and interns from different institutions to understand the various underlying facts about dental pain and its management.

### What is known about this topic

- Self-medication may lead to serious consequences, especially in pediatrics, geriatrics, pregnancy and lactation;
- The prevalence of self-medication among medical students is very high;
- Analgesics are the most common drugs used for self-medication.

### What this study adds

- The findings indicated that 79% had diagnosed their dental pain on their own;
- The results showed that majority of students were depending on internet to diagnose their dental problem and treat it accordingly;
- Forty two percent of students affirmed that they were confident about managing their symptoms by any method without consulting a dentist.

## Competing interests

The authors declare no competing interests.
